# Veritable Untruths: Autistic Traits and the Processing of Deception

**DOI:** 10.1007/s10803-021-05347-4

**Published:** 2021-11-18

**Authors:** Wei Li, Hannah Rohde, Martin Corley

**Affiliations:** 1 Psychology, PPLS, University of Edinburgh, 7 George Square, Edinburgh, EH8 9JZ UK; 2 Linguistics and English Language, PPLS, University of Edinburgh, Dugald Stewart Building, Edinburgh, EH8 9AD UK

**Keywords:** Disfluency, Autism spectrum quotient, Eye-tracking

## Abstract

How do we decide whether a statement is literally true? Here, we contrast participants’ eventual evaluations of a speaker’s meaning with the real-time processes of comprehension. We record participants’ eye movements as they respond to potentially misleading instructions to click on one of two objects which might be concealing treasure (*the treasure is behind thee, uh, hat*). Participants are less likely to click on the named object when the instructions are disfluent. However, when hearing disfluent utterances, a tendency to fixate the named object early increases with participants’ autism quotient scores. This suggests that, even where utterances are equivalently understood, the processes by which interpretations are achieved vary across individuals.

## Introduction

Even though autism spectrum disorder (ASD) is generally associated with a deficit in social and figurative aspects of communication, many previous studies investigating the abilities of participants with ASD to understand non-literal language have shown that these participants obtain similar comprehension scores to their neurotypical peers (e.g., Happé, 1994, 1995; Hala et al., 2007; Jolliffe & Baron-Cohen, 2000; Loukusa & Molianen, 2009). This raises the question of whether individuals with ASD and those without achieve similar outcomes by employing different processes. Here, we develop the theme of processing differences, with a focus on how the manner of a spoken message is delivered, in particular, disfluent fillers such as “um” and “uh”, influences individuals’ comprehension.

Fillers are often regarded as socially oriented, influencing discourse dynamics by controlling the flow of conversation (e.g., Clark & Fox Tree, 2002), signalling hedges in conversation (Smith & Clark, 1993), or being taken as cues to deception (Loy et al., 2016; Vrij & Semin, 1996; Zuckerman et al., 1981). Previous studies have shown that high-functioning individuals with ASD produce fewer fillers than do the typical population (Lake et al., 2011). Children with autism produce fewer fillers compared to their typically developing peers (Gorman et al., 2016; Heeman et al., 2010; Irvine et al., 2016). This reduced use of fillers, considered as a conversational cue, is consistent with a characterization of speakers with ASD as being less able to take their listeners’ perspectives (Colle et al., 2008; Paul et al., 2009). A decreased attention to social cues may yield difficulty in understanding the implicit social meanings associated with fillers (Irvine et al., 2016).

However, this difficulty may not manifest in comprehension outcomes. Participants who struggle with social cues may nonetheless find ways to interpret fillers in an equivalent way to their peers. By focusing on the outcome of comprehension, usually operationalised as whether the eventual interpretation of an utterance aligns to a norm, it is possible that differences in the processes which underlie these outcomes may be overlooked (McKenna et al., 2015). McKenna et al. (2015) demonstrated a difference in the processing of a type of figurative language, metonymy, in which an attribute of a concept instantiates that concept (such as the use of *Dickens* when referring to the books written by Charles Dickens rather than to the writer himself). In their study, participants without diagnoses completed an autism spectrum quotient questionnaire (AQ: Baron-Cohen et al., 2001) and read sentences containing target nouns (e.g., *Vietnam, Finland*), in contexts supporting literal or figurative, metonymic, interpretations (e.g., *During my trip, I hitchhiked around Vietnam/Finland; A lot of Americans protested during Vietnam/Finland*). Their results showed an “unfamiliar metonym disadvantage” where participants were slower to read a novel metonym (e.g., *Finland*) in its figurative sense, compared to a metonym which has its figurative meaning established in daily use (e.g., *Vietnam* referring to the conflict during the 1950s–1970s). Of relevance to the present study, they found that participants with higher scores on the autism quotient measure were relatively slowed down by these novel metonymies, while those with lower numbers of self-reported autistic traits were not. Note that all participants scored 100% on comprehension scores: differences related to AQ could not be found in any measure of interpretation, but were instead only evident when the time course of the processing of the stimuli was investigated.

Although understanding figurative meanings in written language, such as that investigated by McKenna et al. (2015), can be difficult, understanding non-literal meaning in speech adds additional complexity, because artefacts such as fillers can modify literal meaning. Loy et al. (2017) investigated whether interpretations of a sentence (truthful or deceptive) made by listeners in the general population could be influenced by disfluency. Their experiments used a lie-detection game setting, where participants were presented with two images (named image/distractor image) in every trial and were asked to click on the image that they thought concealed some treasure. For each trial, participants heard a pre-recorded fluent or disfluent utterance describing the location of the treasure. Participants’ image clicks revealed a bias to interpret disfluent speech as a cue to speaker deception about the location of the treasure. Importantly, eye movements were also measured, and disfluent speech resulted in more frequent early eye movements towards the distractor item, showing the influence of an early process associating disfluency with deception.

Loy et al. (2017) study provides two essential points of reference for the current study. First, the method allows us to directly observe the time-course of disfluency comprehension, providing a way to investigate how the manner in which something is said might affect its ongoing interpretation. Second, along with other situations which depend on understanding the interlocutor’s intentionality, the recognition of deception has been shown to be difficult for individuals with ASD: for example, it is harder for children with autism to lie to others and to detect when they are being lied to (Ranick et al., 2013). By using a lie detection paradigm in which disfluency can serve as a potential pragmatic cue, we can examine in detail any differences in the processes of comprehension that might arise between individuals. Specifically, we can measure both participants’ eventual interpretations—truth or deception?—and the real-time processes that precede that outcome.

Our experiment replicates experiment 2 from Loy et al. (2017), with a set of participants from the general population who vary in their autism spectrum quotients (AQ). AQ is not a diagnostic test of ASD, but it is used to assess broader autism phenotype (BAP) metrics for understanding subclinical ASD traits within individuals in the general population who are presumed to have typical developmental profiles. The test comprises 50 questions focusing on five BAPs: social skills, attention switching, attention to detail, imagination, and communication (Baron-Cohen et al., 2001). AQ scores from low to high quantify where a given participant is situated on a putative continuum from neurotypical to autism diagnosis (Baron-Cohen et al., 2001). While our findings do not allow us to draw direct conclusions about comprehension processes in ASD, the study emphasizes the utility of real-time measures in comparing processing differences among populations that vary on an AQ spectrum.

Our experiment measures the influence of manner of delivery on pragmatic comprehension in two ways. First, we measure the *outcome* of the comprehension process by recording which object people click on. This gives us a direct indication of whether a participant has interpreted a given utterance as truthful, in which case they are likely to click on the named (target) object, or deceptive, in which case they are likely to click on the other (distractor) image. If the results of Loy et al. (2017) are replicable, we expect to see an effect of the presence of disfluency, such that participants are less likely to click on the target following a disfluent utterance. We further assess the *process* of comprehension. By measuring participants’ eye movements time-locked to the speaker’s mention of the target object, we can test the time-course of comprehenders’ responses to the speaker’s manner of delivery. If the results replicate those of Loy et al., we should see a decrease of bias towards the named object in the disfluent condition. Of critical interest in the present study is whether the outcome or process measures interact with AQ. An interaction at the outcome level (item clicked) would suggest that the levels of broader autism phenotypes measured by the AQ affect the eventual understanding of what is said. Regardless of whether such an interaction is found or not, an interaction at the process level (eye movements) would suggest differences in the processes underlying non-literal interpretations, opening the door to research with formally-diagnosed groups of participants.

## Methods

### Participants

Ethical approval was given by the PPLS Research Ethics Committee with reference number 100-1920/1. Sixty-two self-reported native English speakers (Male: 18; Female: 44) took part in the experiment. Participants ranged from 18 to 35 years old, mainly comprising students at the University of Edinburgh recruited from a wide variety of disciplines, including Engineering, Chemistry, Mathematics, Law, and Psychology. Participants were paid £5 for participation. Participants had normal or corrected-to-normal vision, and all used their right hands to control the mouse used in the experiment. Participants provided written consent prior to the beginning of the experiment.

### Material

#### Autism Spectrum Quotient

The autism spectrum quotient (AQ: Baron-Cohen et al., 2001) questionnaire comprises 50 items reflecting five different broader autism phenotypes either positively (e.g., “I am fascinated by numbers”, “I am often the last to understand the point of a joke”), or negatively (e.g., “I find social situations easy”). Participants are asked to respond based on a 4-point Likert scale varying from “definitely agree” to “definitely disagree”. Each response represents one point; and a participant’s AQ score is calculated by totalling their responses (taking polarity into account), with a maximum score of 50. Studies to date suggest that individuals diagnosed with autism tend to score 32 or more, compared to individuals without autism who tend to average around 16. However, the AQ is not a diagnostic test, and autistic traits are present in the wider community (see Folstein & Rutter, 1977; Persico & Bourgeron, 2006 for details). Therefore, AQ scores should be interpreted very carefully. Participants in the current study were informed of this beforehand and were told that their AQ scores would not be provided to them.

#### Visual Stimuli

The visual world paradigm from Loy et al. (2017, experiment 2) was adapted for the current study. Visual stimuli included 130 line-drawings from Rossion and Pourtois (2004), which were grouped into pairs across 65 trials (5 practice trials; 20 critical; 40 fillers). For each pair of drawings in each trial, the drawing that the speaker named as concealing the hidden treasure is referred to as the *target* picture, and the other one the *distractor* picture. The two pictures were presented vertically centred on the screen, and horizontally centred at 25% (left-hand picture) and 75% (right-hand) of the screen width. The target picture’s position on the left versus the right was counterbalanced across items.

#### Audio Stimuli

The audio files used in the current experiment were taken from Loy et al.'s Experiment 2. Participants were told, as a cover story, that the speaker had taken part in an earlier experiment, in which she had been told to try and mislead her partner about the treasure’s location by sometimes lying.

In 20 critical trials, the recordings of the speaker describing the purported locations of the ‘treasure’ were categorized as either *fluent* (e.g., “The treasure is behind the [target]”) or *disfluent* (e.g., “The treasure is behind thee, *uh*, [target]”). To ensure that participants were exposed to the same utterance (bar disfluency) across conditions, and the same disfluency across disfluent trials, complete fluent and disfluent sentences were first recorded, and then a prolonged article followed by a filler cut out from a disfluent utterance was spliced into each relevant fluent utterance to create the corresponding disfluent version. Disfluent utterances were therefore characterized by a prolonged article (“the” pronounced /ðiː/, or “thee”) and the disfluent segment (i.e., “uh”) before the target noun.

The assignment of fluent/disfluent conditions to items was counterbalanced across two lists. However, due to a coding error, one critical item (“lamp”) in List 1, which should have been in the fluent condition, was mistakenly paired with a disfluent recording. Thus, the final numbers of items in experimental conditions for participants using audio files from List 1 were n = 9 (fluent items) and 11 (disfluent items).

To obscure the experimental manipulation, and reinforce the impression that participants were listening to natural recordings, forty additional filler trials referred to pairs of images that were not used in critical trials, and were designed such that half included disfluencies other than “uh”, or discourse manipulations such as discourse markers (e.g., “*Okay*, the treasure is behind the [target]”) and modals (e.g., “The treasure *could be* behind the [target]”), and the other half were fluent.

### Procedure

The experiment was in two parts, a computer-based lie detection game and an Autism-Spectrum Quotient (AQ) measurement. The researcher began by introducing the process of the whole experiment and the lie detection game task. The eye-tracking experiment which followed was presented using OpenSesame version 3.2.7 (Mathôt et al., 2012), with movements of the right eye monitored using an Eyelink 1000 Tower Mount system. Participants were seated at a viewing distance of 80 cm from a 19″ CRT monitor. They read the instructions first which explained that their aim was to collect as much treasure as possible by clicking on the object concealing the treasure on each trial.

Following reading the brief instructions, a press of the spacebar started the calibration of the eye-tracker. Five practice trials followed the calibration; at the end of the practice session, participants pressed the space bar to commence the sixty experimental trials (20 critical trials and 40 fillers).

**Fig. 1:**
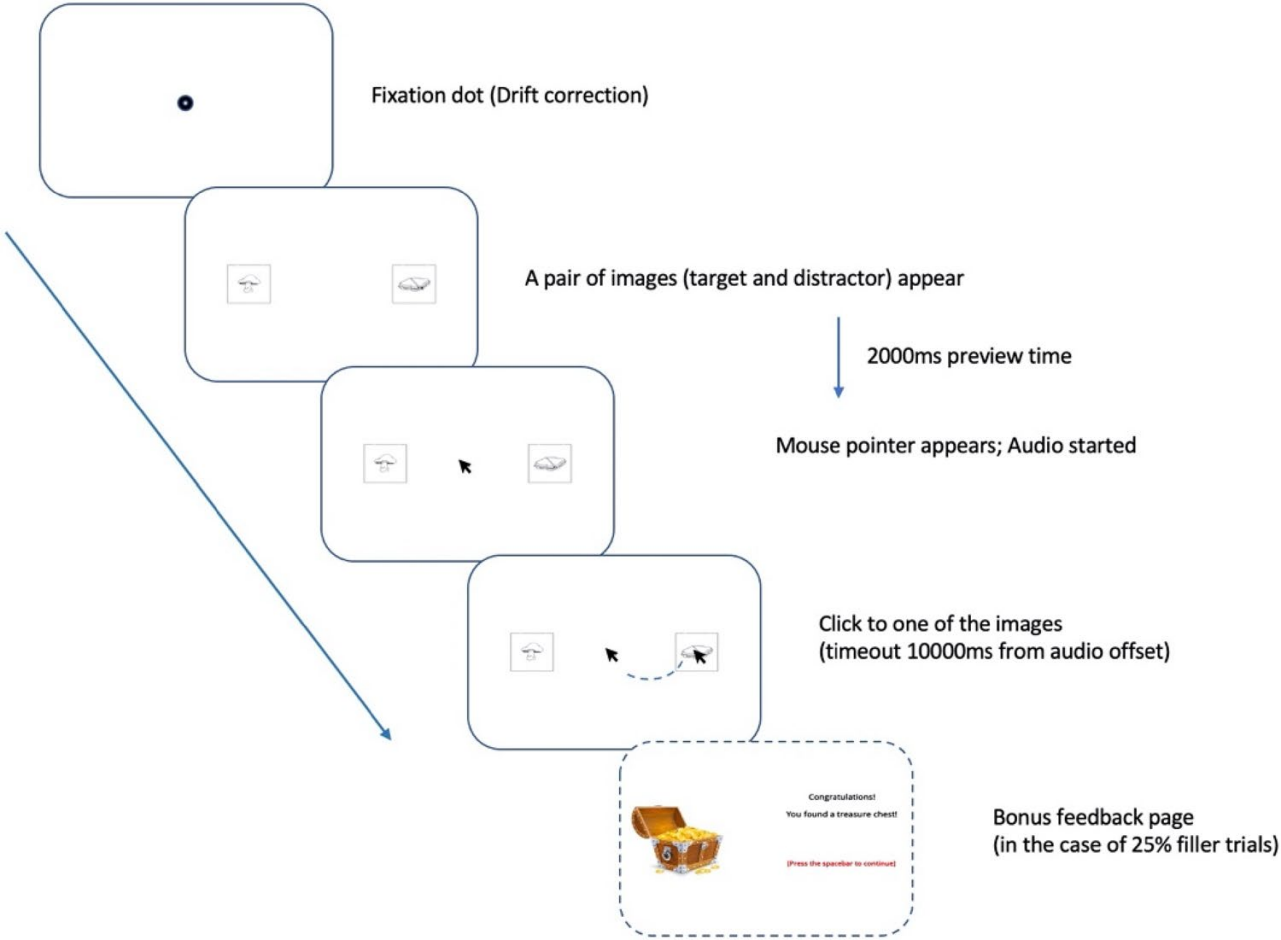
Procedure of a single trial from the experiment

Each new trial began with a black fixation dot at the centre of the screen, and participants were told to carefully stare at the black dot when they were ready to start a trial, allowing a drift correction to be applied if necessary. As soon as the fixation dot disappeared, a pair of images—a target and a distractor—appeared on the screen. After a preview period of 2000 ms, the mouse pointer appeared at the centre of the screen, and the audio stimulus started. Participants were instructed to click on the object which they believed the treasure was behind as soon as they could. Once one object had been clicked on, the images disappeared, providing visual feedback that the click had been recorded. 1000 ms after the end of the audio, or once the participant had clicked on an object—whichever occurred later—a black dot appeared which indicated the onset of the next trial, except in the case of 25% of filler trials, which included a bonus feedback page after clicking (see detailed explanation below). Figure 1 shows the complete flow of one example trial from the experiment.

Three aspects of the experiment were designed to ensure that participants remained motivated to discover the “true” location of the treasure throughout. The first is that participants were informed that their score would be counted based on how much treasure they managed to find, and that good scores would be entered on a high-score table which was shown at the end of the experiment. Second, participants were told that there were a certain number of bonus trials, from which they could earn more points than from other trials. To this end, 25% of the filler trials were immediately followed by a screen saying that a bonus treasure chest had been found, regardless of which picture had been clicked. To prevent learning, immediate feedback, such as this bonus message, was only provided in a small number of the filler trials, not in any critical trial, and only to keep participants motivated. Third, if no click was detected within 5 s of audio onset, a time-out message would be automatically shown on the screen to encourage participants to respond more quickly.

Eye movements were recorded during all experimental trials, together with the final object the participants clicked on. Only the data from critical trials were analysed.

Once the treasure-hunting part of the experiment had finished, participants filled out a paper version of the AQ test (Baron-Cohen et al., 2001). Finally, participants were fully debriefed about the aims of the experiment.

## Results

Data and analysis scripts are available at https://osf.io/jwhfr/. Prior to analysis, practice and filler trials were removed, as well as the trials in which participants failed to click on either object within 5000 ms of audio onset (0.5% of the critical data).

### Autism Quotient

Across 62 participants, AQ ranged from 2 to 38, with a mean of 15.4 (SD 7.26) and a median of 15.0. For illustrative purposes only, participants were split at the median into two equal-sized groups, respectively referred to as the Low- and High-AQ groups. Characteristics of the participants in each group are shown in Table 1, and these groups will be used in tables and figures to illustrate effects.

**Table 1:** Characteristics of High-AQ group and Low-AQ group

	Mean AQ (SD)	Mean age (SD)	Gender
Low-AQ group	9.74 (3.04)	23.00 (0.5)	24F/7M
High-AQ group	21.16 (5.53)	22.10 (0.5)	20F/11M

### Final Object Clicks

Final object-click results were determined by two factors: whether the x-coordinate of the mouse when clicking the object is greater than 0, and the position of the target in that trial (such that if x > 0 and the target is on the right, the target has been clicked). The percentages of trials in which participants clicked on targets and distractors, for the whole study and for the High- and Low-AQ participant groups, are given in Table 2.

**Table 2:** Percentage of mouse clicks recorded on each object (target/distractor) by manner of delivery (fluent/disfluent), for the whole study

	Whole study	Low-AQ	High-AQ
**Fluent**			
Target	66.4	67.0	65.9
Distractor	33.6	33.0	34.1
**Disfluent**			
Target	50.2	50.3	50.2
Distractor	49.8	49.7	49.8

The binary outcome of clicking on the target versus distractor was modelled in a mixed-effects logistic regression with one within-participants predictor of manner of delivery (fluent or disfluent) and one between-participant predictor of (raw, centred) AQ score, including random intercepts and a slope for manner of delivery by participant, as well as random intercepts by target image. Participants were 2.71 times more likely to click on the target following a fluent utterance compared to a disfluent utterance (β = 0.9966, SE = 0.3052, *p* = 0.0011). There were no effects of AQ (β = − 0.0226, SE = 0.0256, *p* = 0.3784), or interactions between fluency and AQ (β = 0.0121, SE = 0.0421, *p* = 0.7743).

### Eye Movement

**Fig. 2:**
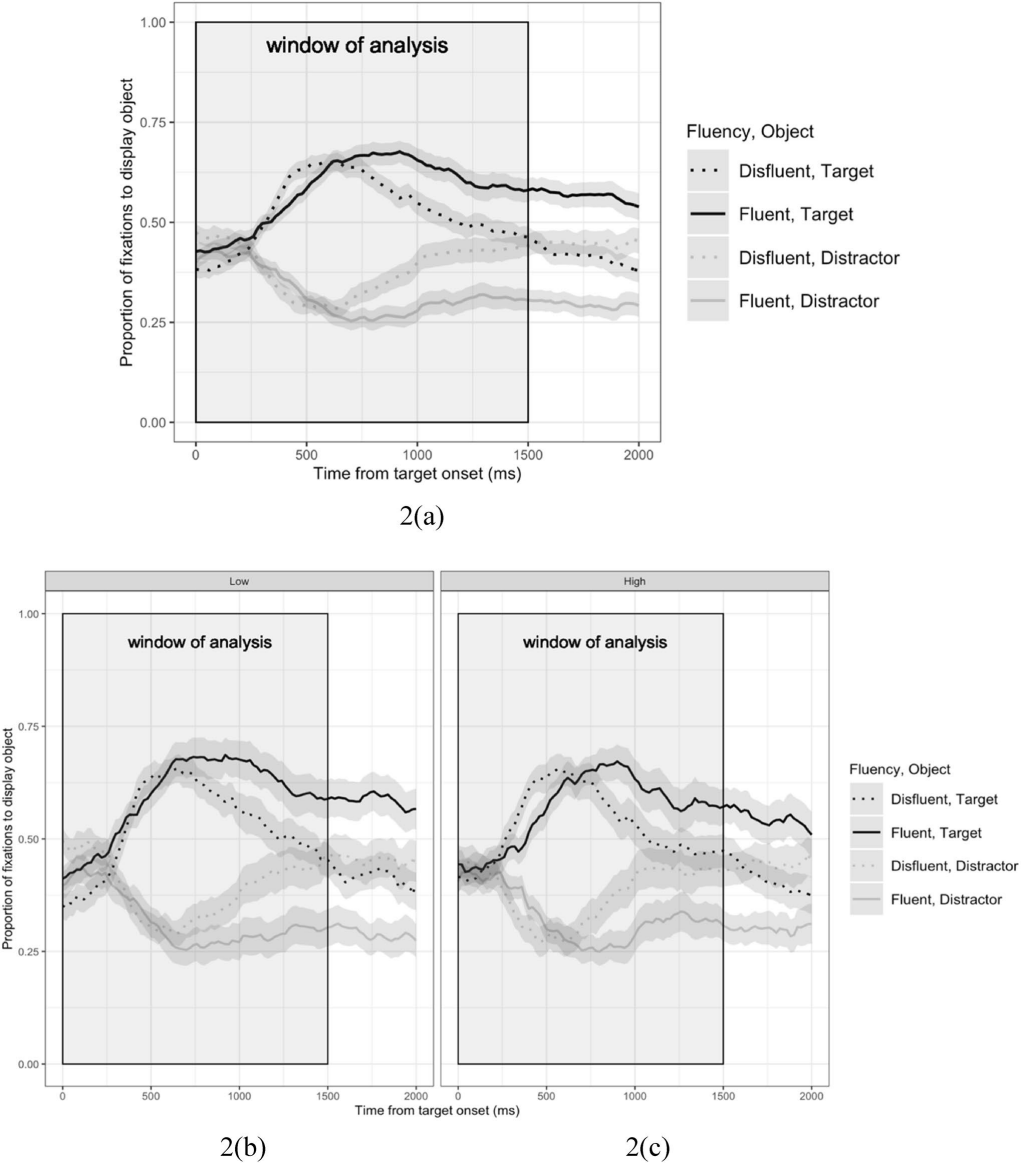
Mean proportions of fixations to target object and distractor over 2000 ms from target onset, for fluent and disfluent conditions for all participants (2a), low- (2b) and high-AQ (2c) groups, calculated from summed fixations for each 20 ms time bin from target onset to 2000 ms post-onset. The shaded rectangular area represents the analysis window from target onset to 1500 ms later. The shaded areas represent ± 1 standard error of the mean by items

Measuring the frequencies over time with which participants fixate each of the images presented, using the eye tracking record, allows us to investigate how biases are updated in the ongoing interpretation of what is said (see Cooper, 1974; Tanenhaus et al., 1995 for more details). In the present experiment, fixation data were averaged into bins of 20 ms (10 samples). The proportions of time participants spent fixating objects were coded according to region of interest (target/distractor) for each bin. Figure 2a shows the time course of all participants’ fixations to target objects and distractors over 2000 ms (100 bins) starting at target onset, for fluent and disfluent conditions respectively. For illustrative purposes, Fig. 2b, c show the equivalent patterns for the Low- and High-AQ groups of participants (Fig. 2).

**Fig. 3:**
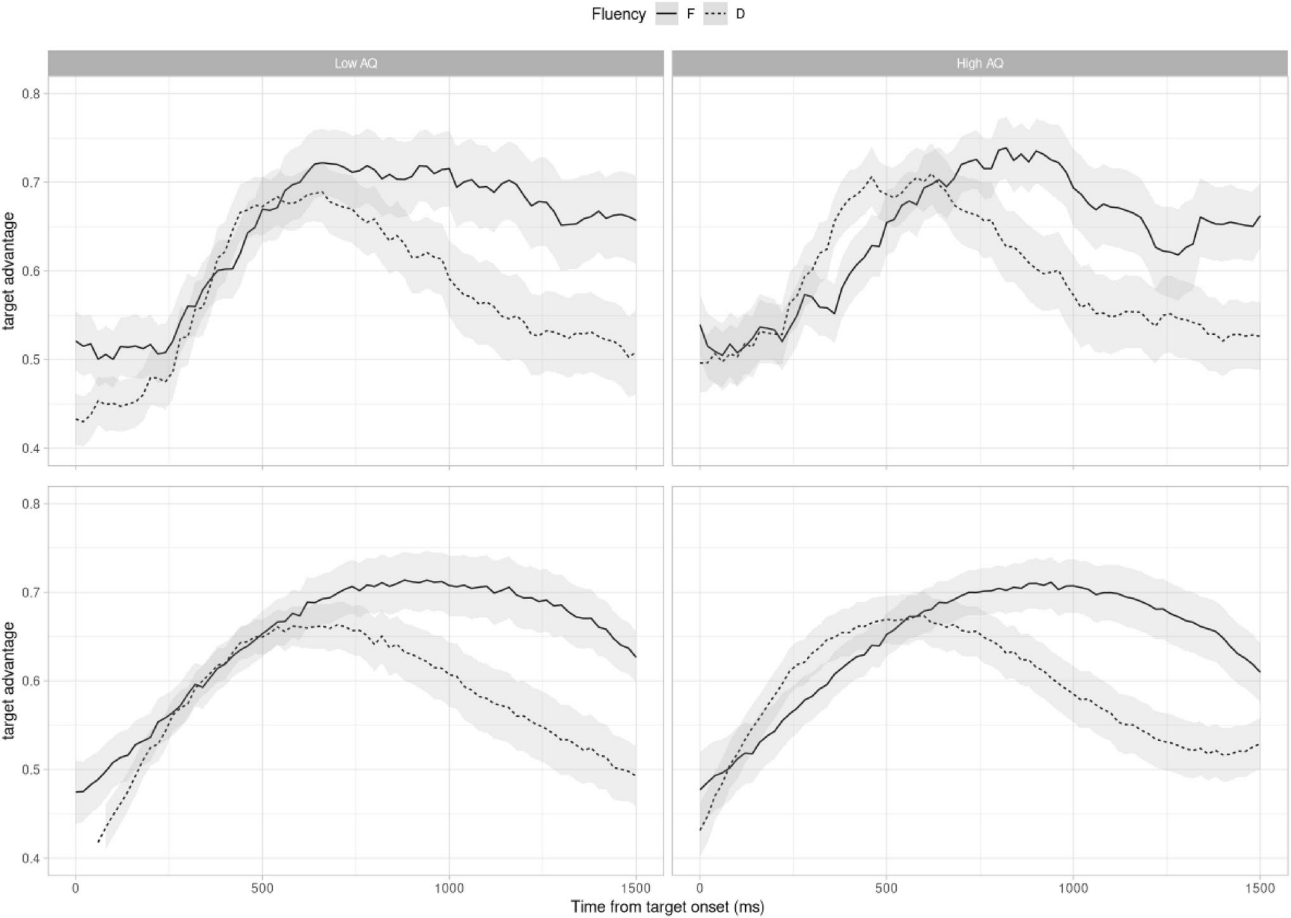
Recorded (top) and model-predicted (bottom) target advantage, for Low-AQ (left) and High-AQ (right) groups of participants. Model predictions are derived from 101 posterior samples from the converged model

We analysed eye movement data over a time window which began at target onset and ended 1500 ms later. This window was chosen based on previous research suggesting that the resolution of a referent is normally established around 400-800 ms after the object being mentioned (Eberhard et al., 1995; Hanna et al., 2003), with additional time allowed to capture any potential differences in later processing. For the purposes of analysis, we calculated a binomial “target advantage” for each bin: bins in which the majority of time was spent fixating the target were coded as 1; bins in which the majority of time was spent fixating the distractor were coded as 0. Bins in which neither target or distractor were fixated (9354) or in which both images were fixated equally (21) were discarded (10% of the data). Figure 3 shows target advantage, split by High- and Low-AQ groups, for reference.

We constructed a Bayesian generalised linear mixed model, with a logit link function, to analyse our data. We fit a model including orthogonal 1st-3rd order polynomial representations of time, allowing us to properly capture the non-linear nature of the target advantage shown in Fig. 3. As well as the time variables, the model included a within-participants and -items effect of disfluency; and a between-participants, but within-items, measure of (scaled) AQ, as well as interactions between disfluency, AQ, and each of the polynomial orders of time. Random effects for intercepts and slopes (but not interactions) were included. The model was fit using the brms package in R (Bürkner, 2017), version 2.16.1, using default priors, with four chains of 4000 iterations each. The Rhat parameter for each parameter in the model was equal to 1, indicating successful model convergence; visual inspection of posterior predictive checks suggested a good fit to the data.

Details of the converged model are given in Table 3. For each effect we report a mean effect estimate, the upper and lower bounds of a 95% credible interval, and the probability that a given effect is different from zero in the direction of its sign. There is evidence for an effect of disfluency (the probability that participants fixate the target less overall when the utterance is disfluent is 0.986) and there are notable interactions of disfluency with the polynomials of time (effectively, indicating that the curve over time of the target advantage has a different shape when utterances are disfluent). Critical to the present paper are the three-way interactions between AQ, Fluency, and each of the polynomial orders of time. There is strong evidence for each interaction (all *p*s ≥ 0.992), showing that the target advantage curve changes with AQ, such that participants with higher AQ scores tend to show earlier biases towards the target. Figure 3 shows posterior predictions from the Bayesian model for the Low- and High- AQ groups, to illustrate this effect (but it is important to note that the effect is continuous, across the range of reported AQs).

**Table 3:** Model estimates for eye-tracking data, showing mean effect estimates in logits, upper and lower bounds of 95% credible intervals, and probabilities that effects differ from zero in the direction of their signs (based on posterior sampling)

Effect	b	L95%	H95%	*p* (b > / < 0)
(Intercept)	0.76	0.51	1	–
AQ	− 0.12	− 0.34	0.10	0.863
Disfluency	− 0.33	− 0.64	− 0.03	0.986
Time	2.53	1.21	3.85	1
Time^2^	− 2.13	− 3.09	− 1.23	1
Time^3^	− 0.54	− 1.23	0.13	0.944
AQ:Disfluency	0.02	− 0.23	0.27	0.552
AQ:Time	− 0.46	− 1.56	0.61	0.802
AQ:Time^2^	0.08	− 0.55	0.71	0.590
AQ:Time^3^	0.12	− 0.41	0.67	0.671
Disfluency:Time	− 2.36	− 2.63	− 2.09	1
Disfluency:Time^2^	− 0.42	− 0.70	− 0.15	0.999
Disfluency:Time^3^	1.36	1.09	1.63	1
AQ:Disfluency:Time	− 0.61	− 0.88	− 0.33	1
AQ:Disfluency:Time^2^	0.34	0.07	0.62	0.995
AQ:Disfluency:Time^3^	0.33	0.07	0.60	0.992

## Discussion

The results reported here confirm that listeners’ pragmatic judgements are influenced by manner of speech, and that disfluent instructions are associated with deception. We took the greater frequency of clicks on the distractor object after hearing an instruction such as “The treasure is behind, thee, uh, hat” as evidence that participants were less inclined to believe the location of the treasure named by the speaker when the utterance was disfluent. Moreover, eye movements made during the early time-course of comprehension showed that these judgements were made quickly. These findings replicate the results of Loy et al. (2017).

Note that in these studies, participants were explicitly told that they might be deceived, and the utterances to which they responded were recorded. Whether there are general conclusions to be drawn about deception is unclear; here we are concerned with the processing of language that is ‘known’ not to have a literal interpretation. We can however draw some ecological comfort from a study by Loy et al. (2018), in which participants’ judgements were affected in a similar way by disfluency in a two-player version of the current deception game, in which all utterances were spontaneous.

Critically, the present study shows that the general effects of disfluency described above vary by AQ. When hearing disfluent instructions, participants with higher AQ scores, and therefore higher numbers of traits typically associated with autism, are more likely to make early fixations on the target. The evidence for these effects is manifest as a series of interactions with a continuous AQ score predictor, and therefore not subject to criticisms typically applied to median splits (e.g., McClelland et al., 2015). However, the differences emerge as differences of processing but not of comprehension: our data do not suggest that participants’ final interpretations vary with AQ, but they do suggest that there is variation in the mental processes which underlie those interpretations.

A potential account of these findings is that as the number of autistic-like traits associated with higher AQ scores increases, participants have more difficulty in utilizing contextual information. This may lead to a weaker association between disfluency and deception, attenuating the early tendency to fixate the distractor following a disfluent utterance. A variant of this account is that it may be more difficult to override more basic effects of filler disfluencies, such as their tendency to focus listeners’ attention on the upcoming message (Collard et al., 2008; Fox Tree, 2002). According to the latter view, participants with higher AQs may show an initial tendency to fixate the target quickly following a disfluency, before this is overridden by slower pragmatic reasoning.

It is important to stress again that these findings are only relevant to people diagnosed with autism to the extent that AQ measures can be considered a proxy for diagnosis. The critical aim of the paper is not to identify a processing difference in the clinical population, but to point to a likely candidate which merits further investigation. Moreover, because of the continuous nature of the AQ, some statistical issues which can arise from group comparisons (especially when “controlling” for other varying factors: Miller & Chapman, 2001) are avoided. We can be confident (to the usual limits of statistical inference) that the effect is real: in the subclinical population, comprehension processes may result in the same outcomes, even though the processes by which those outcomes are achieved may vary.

In line with the study by McKenna et al. (2015), the implication of the current study is that by merely focusing on the outcomes of cognitive processing, it is possible to miss the real differences in detailed processes that vary across the population. Previous studies have used tasks in which participants are asked to provide context-appropriate explanations for non-literal speech, such as Happé’s Strange Stories Test (Happé, 1994), in exploring disfluency comprehension in ASD children. However, many of these studies represent cases in which ASD- and non-ASD individuals achieved similar results, despite the ASD participants being expected to show less developed ability (Hala et al., 2007; Happé, 1995; Jolliffe & Baron-Cohen, 2000). Such results may not be conclusive without exploring the detailed cognitive processes underlying any parity in behavioural outcomes.

The present study suggests that there are differences in the processing of disfluency among individuals varying in numbers of self-identified autistic traits. This may, by extension, have implications for the comprehension processes of people with ASD diagnoses.
